# Association of Korean Medicine and polypharmacy with fall risk and mortality in older adults with stroke

**DOI:** 10.3389/fphar.2025.1621819

**Published:** 2025-06-19

**Authors:** Ye-Seul Lee, Bo-Hyoung Jang, Jin Pyeong Jeon, Han-Gyul Lee, Seungwon Kwon, Woo-Sang Jung

**Affiliations:** ^1^ Jaseng Spine and Joint Research Institute, Jaseng Medical Foundation, Seoul, Republic of Korea; ^2^ Department of Preventive Medicine, College of Korean Medicine, Kyung Hee University, Seoul, Republic of Korea; ^3^ Department of Neurosurgery, Hallym University College of Medicine, Chuncheon, Republic of Korea; ^4^ Kyung Hee University Medical Center, Department of Cardiology and Neurology, College of Korean Medicine, Kyung Hee University, Seoul, Republic of Korea

**Keywords:** polypharmacy, Korean medicine, stroke, falls, mortality

## Abstract

**Background:**

Stroke survivors often take multiple medications (polypharmacy), raising concerns about falls and mortality in older adults. This study investigated whether Korean Medicine (KM)—primarily acupuncture—is associated with fall risk and mortality among older adults with stroke and polypharmacy.

**Methods:**

A population-based retrospective cohort study using South Korea’s National Health Insurance Service (NHIS) claims database. Adults aged 65 or older with a first stroke in 2015 were included if they had five or more prescribed medications (polypharmacy) or ten or more (hyper-polypharmacy) for at least 270 days. KM users received acupuncture or electroacupuncture (≥3 outpatient visits or ≥1 inpatient stay) within a year of stroke onset. The primary outcome was falls resulting in fracture; the secondary outcome was all-cause mortality—both assessed over 3 years. Propensity score matching balanced demographics, comorbidities, and medication use. Cox proportional hazards and subgroup analyses were conducted. Subgroup and sensitivity analyses explored effect modification.

**Results:**

Among 25,034 older stroke patients, 10,011 had polypharmacy; of those, 6,809 used KM. After matching, 3,127 KM users were compared with 3,127 non-users. KM users with polypharmacy had a higher rate of falls but lower all-cause mortality than non-users. In hyper-polypharmacy, KM use did not significantly affect falls but was associated with lower mortality. Sensitivity analyses of the unmatched cohort, alternative outcome definitions, and interactions yielded consistent patterns.

**Conclusion:**

In older adults with stroke and polypharmacy, KM may improve functional recovery and mobility, potentially increasing falls if balance training is inadequate, yet simultaneously confer survival advantages—perhaps through neuro-immune or systemic effects—irrespective of medication load. Among the more frail hyper-polypharmacy group, KM reduced mortality without altering falls, suggesting that functional gains and competing-risk dynamics differ by medication intensity. Prospective studies with granular functional measures, drug–drug interaction data, and formal competing-risk models are needed to optimize the safe integration of KM into comprehensive stroke care.

## 1 Introduction

Polypharmacy—taking more medications than clinically necessary—usually refers to the use of five or more drugs (Kim et al., 2014; Masnoon et al., 2017). With population aging and the rising prevalence of multiple chronic conditions, polypharmacy has become a growing global concern, with about half of those aged 65 and older taking five or more medications (Wastesson et al., 2018). In South Korea, 86.4% of older adults take six or more medications, and 44.9% are in a state of excessive polypharmacy by taking 21 or more medications (Kim et al., 2014). Older adults with polypharmacy face a heightened risk of adverse events, including cognitive impairment, falls, and mortality (Gnjidic et al., 2012; Kojima et al., 2012b). Potentially inappropriate medications often contribute to this problem by negatively affecting cognitive function and elevating the likelihood of neurodegenerative diseases (Park et al., 2017a; Park et al., 2017b).

Stroke survivors are particularly vulnerable to polypharmacy. As stroke commonly co-occurs with hypertension, diabetes, dyslipidemia, and heart disease, many patients take multiple drugs to address both risk factors and symptoms (Kose et al., 2017). A Scottish study reported that patients with stroke were more than twice as likely to have multimorbidity than those without stroke, with 12.6% taking 11 or more medications, compared with only 1.5% of the general population (Gallacher et al., 2014). Indeed, patients with stroke on more than five or six medications face an elevated risk of adverse events and falls (Kojima et al., 2012a; Kojima et al., 2012b). Given the physical disabilities and reduced quality of life often associated with stroke, polypharmacy can further complicate recovery and create additional barriers to rehabilitation.

Korean Medicine (KM), a branch of Traditional East Asian Medicine (TEAM), has a long history alongside Chinese and Kampo medicine (Lee et al., 2014). In recent years, TEAM approaches have been investigated for their potential to reduce polypharmacy. One retrospective study of 159 patients reported a significant drop in the total number of medications used after treatment with TEAM in those who showed symptomatic improvement (Takano et al., 2018). Another case report documented a reduction from 13 to two medications alongside clinical improvement (Nogami et al., 2019). These findings suggest that by addressing the symptoms that originally led to multiple prescriptions, TEAM—including KM—may lessen the medication burden. However, prior investigations have focused mainly on drug count reduction rather than exploring the broader impact on falls and mortality.

We utilize data from the National Health Insurance Service (NHIS) to examine whether insurance-covered KM treatments, namely acupuncture, can influence polypharmacy-related outcomes in older patients with stroke. Specifically, we compare fall and mortality rates among those with and without polypharmacy and KM usage. By clarifying KM’s potential role in mitigating the risks associated with multiple medications, we aim to inform more integrative approaches to managing the complex care needs of older stroke survivors.

## 2 Methods

### 2.1 Data source

We utilized the health claims database maintained by the NHIS in South Korea, which includes data for 97.0% of the population (Sun-Min Kim, 2020). The remaining 3.0% of the population is covered by Medical Aid (Kim et al., 2020). Mortality data were obtained from Statistics Korea. The database, along with related materials and metadata, is publicly accessible on the National Health Insurance Data Sharing Service website (Cheol Seong et al., 2017). Access to the database is granted following a review by the NHIS inquiry committee for research purposes. The Institutional Review Board of Kyung Hee University provided a formal waiver for consent [KHSIRB-20-361(EA)].

### 2.2 Study cohort

**FIGURE 1:**
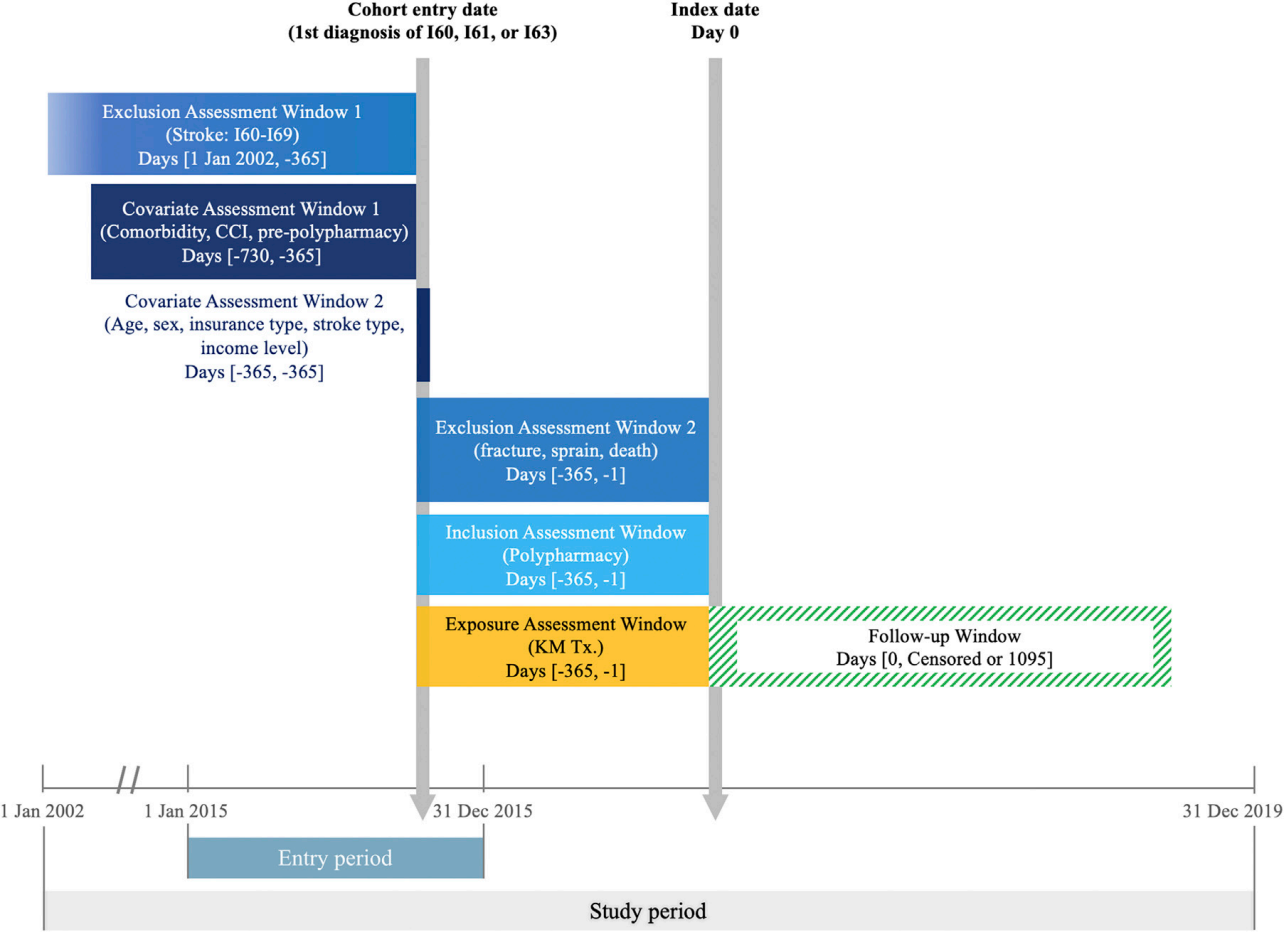
Study design.

Using NHIS claims data, we identified patients diagnosed with cerebral hemorrhage (I60, subarachnoid hemorrhage; I61, intracerebral hemorrhage) or cerebral infarction (I63, cerebral infarction) between January 1 and 31 December 2015, according to the International Classification of Diseases 10th revision. The cohort was restricted to individuals aged 65 and above. Exclusion criteria included patients without a record of hospitalization for at least 1 day and those with a history of any type of stroke (I60–I69) since 2002, the earliest data available from the NHIS. The cohort entry date was determined by the first recorded stroke diagnosis in 2015 (Figure 1).

### 2.3 Polypharmacy

Polypharmacy was defined as the use of five or more medications from a predefined list (Supplementary Table S1) for 270 days or more within the 1-year period following stroke onset (Masnoon et al., 2017; Onoue et al., 2018; Sirois et al., 2019; Chang et al., 2020; Beezer et al., 2021). This particular study was referenced because it utilized the same database (Chang et al., 2020). The use of 10 or more medications from the list of medications for 270 days or more during the same exposure window was defined as hyper-polypharmacy. The list of medications was developed through expert consultations and included 227 drugs commonly prescribed to patients with stroke. These drugs fall into various categories, including cardiovascular agents, anti-Parkinson and anti-dementia agents, psycholeptics and hypnotics (Supplementary Table S1).

### 2.4 Intervention

The intervention of interest was acupuncture and/or electroacupuncture, as documented in NHIS reimbursement records. Patients were considered to have received acupuncture if they had at least three reimbursement records for treatment sessions at a KM outpatient clinic or at least one hospitalization record for treatment at KM hospitals within 1 year of the initial stroke diagnosis.

### 2.5 Outcome and covariates

The primary outcome was the occurrence of various types of fractures within 3 years from the index date. Falls were identified using specific International Classification of Diseases 10th revision (ICD-10) codes: M483–M485, M80, M843, S020–S021, S22–S23, S32–S33, and S72–S73 (Supplementary Table S2). The secondary outcome was all-cause mortality within 3 years from the index date. Patients who experienced falls or died before the index date were excluded from the analysis, as only outcome events occurring after the index date were considered. All patients were followed for 3 years post-index date.

Covariates included baseline demographic factors such as age, sex, and economic status, with economic status determined by annual premiums paid for National Health Insurance in 2015. Predefined comorbid conditions recorded within 1 year prior to the cohort entry date included diabetes mellitus, cancers, chronic back pain, osteoarthritis, rheumatoid arthritis, osteoporosis, chronic obstructive pulmonary disease, dementia, schizophrenia, depressive and anxiety disorders, hyperlipidemia, hypertension, cardiovascular diseases, renal failure, and chronic liver diseases. The Charlson Comorbidity Index (CCI) was also calculated during this period (Supplementary Table S3).

### 2.6 Statistical analysis

Descriptive statistics were utilized to examine the baseline characteristics. Standardized mean differences were calculated for each variable, with an absolute value of ≥0.2 indicating significant imbalances (McCaffrey et al., 2013). Incidence rate ratio (IRR) was calculated for the cohort prior to propensity score matching. A propensity score (PS) representing the probability of receiving KM treatment was calculated using sex, age group, income, types of stroke, pre-stroke polypharmacy, and CCI as classification variables. One-to-one matching was conducted using the greedy nearest neighbor method, with a caliper of 0.1, ensuring exact matches for the abovementioned variables.

Primary analysis focused on assessing the relationship between post-stroke KM treatment and the risk of falls and all-cause mortality among patients with stroke and polypharmacy, using Cox proportional hazards models. A subgroup analysis was conducted for patients with hyper-polypharmacy (10 or more medications). Four prespecified sensitivity analyses were conducted. First, prespecified comorbid conditions were included as a covariate. Second, the number of medications was included as a covariate. Third, the risk of falls and all-cause mortality was estimated for patients with stroke without polypharmacy, or non-polypharmacy (fewer than 5 medications). Fourth, the interaction between KM utilization and medication counts was analyzed. A two-sided p-value of <0.05 was considered statistically significant. All statistical analyses were performed using SAS version 9.4 (SAS Institute, Cary, NC).

## 3 Results

### 3.1 Patient characteristics of polypharmacy cohort

**FIGURE 2:**
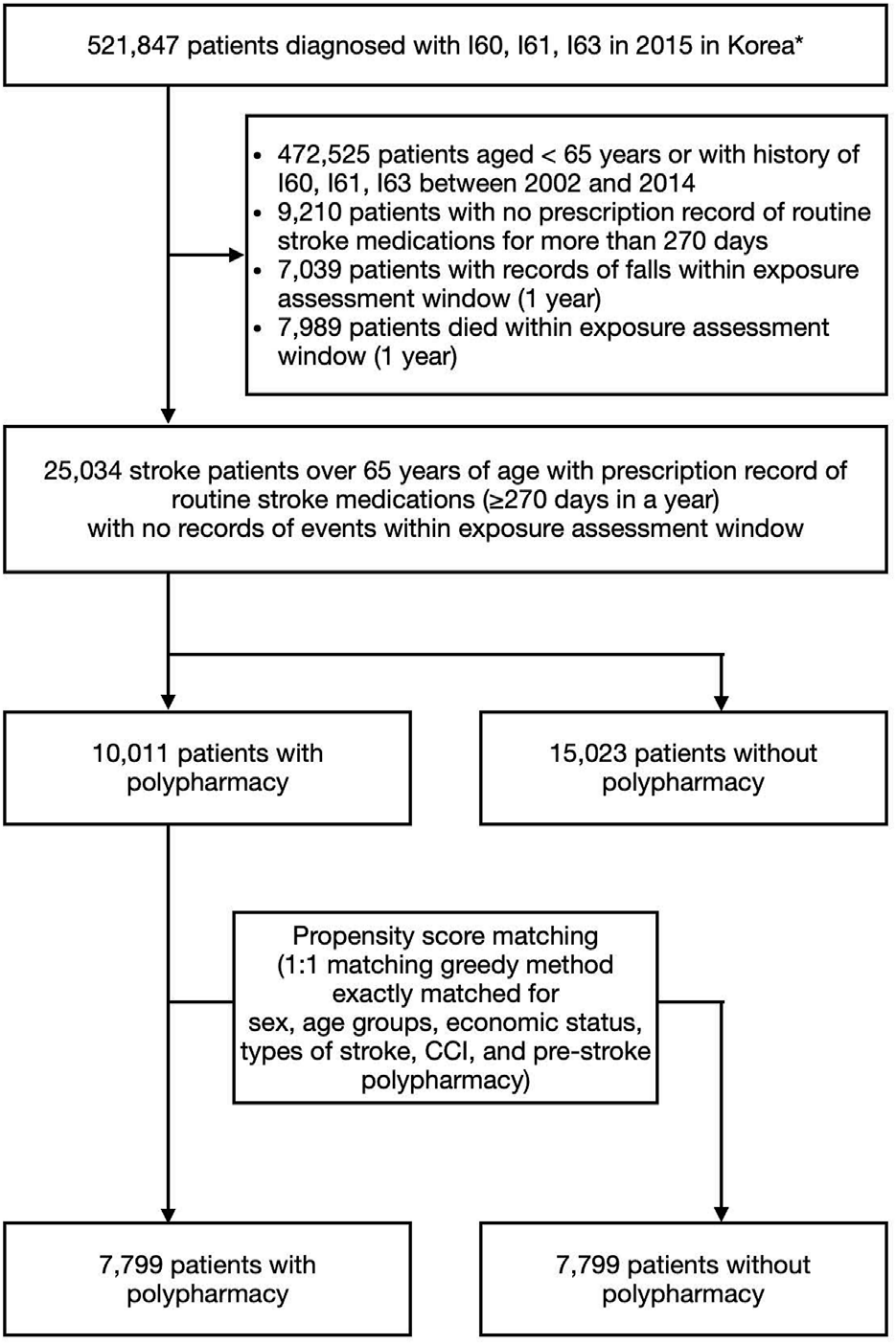
Flowchart of study cohort identification.

The study cohort included 25,034 patients (Figure 2), with 10,011 in the polypharmacy group and 15,023 in the non-polypharmacy group. Within the polypharmacy and non-polypharmacy groups, 6,809 patients (68%) and 10,506 patients (70%), respectively, utilized KM within 1 year following stroke onset. Baseline characteristics revealed that the majority of KM users, regardless of polypharmacy status, were aged 70–79. Additionally, a greater proportion of patients with higher CCI scores, indicative of more severe cases, were among KM users. The distribution of KM use was consistent across various stroke subtypes, including hemorrhagic and ischemic strokes. Patients with pre-existing conditions such as chronic back pain and osteoarthritis also exhibited higher KM use (Table 1).

**TABLE 1:** Baseline characteristics of Korean medicine treatment users and non-users among patients with stroke and polypharmacy.

	Before propensity score matching (*n* = 10,011)			After propensity score matching (*n* = 6,254)		
	KM users (n = 6,809)	Non-users (n = 3,202)	SMD	KM users (n = 3,127)	Non-users (n = 3,127)	SMD
Sex						
Male	3,328 (48.9)	1,898 (59.3)	0.21	1,872 (59.9)	1,872 (59.9)	0.00
Female	3,481 (51.1)	1,304 (40.7)	−0.21	1,255 (40.1)	1,255 (40.1)	0.00
Age groups						
65–69	1,676 (24.6)	750 (23.4)	−0.03	742 (23.7)	742 (23.7)	0.00
70–74	1,894 (27.8)	689 (21.5)	−0.15	686 (21.9)	686 (21.9)	0.00
75–79	1,729 (25.4)	778 (24.3)	−0.03	766 (24.5)	766 (24.5)	0.00
80–84	1,069 (15.7)	602 (18.8)	0.08	599 (19.2)	599 (19.2)	0.00
85–89	362 (5.3)	274 (8.6)	0.13	269 (8.6)	269 (8.6)	0.00
≥90	79 (1.2)	109 (3.4)	0.15	65 (2.1)	65 (2.1)	0.00
Area						
Metropolitan	3,861 (56.7)	1,850 (57.8)	0.02	1,787 (57.1)	1,805 (57.7)	0.01
Rural	2,948 (43.3)	1,352 (42.2)	−0.02	1,340 (42.9)	1,322 (42.3)	−0.01
Economic status						
Low	467 (6.9)	288 (9)	0.08	183 (5.9)	273 (8.7)	0.11
Lower middle	1,111 (16.3)	492 (15.4)	−0.03	490 (15.7)	479 (15.3)	−0.01
Middle	938 (13.8)	456 (14.2)	0.01	433 (13.9)	450 (14.4)	0.02
Upper middle	1,422 (20.9)	675 (21.1)	0.00	668 (21.4)	660 (21.1)	−0.01
High	2,776 (40.8)	1,255 (39.2)	−0.03	1,304 (41.7)	1,231 (39.4)	−0.05
Unknown	95 (1.4)	36 (1.1)	−0.03	-	-	-
Types of stroke						
I60	123 (1.8)	51 (1.6)	−0.02	41 (1.3)	41 (1.3)	0.00
I61	313 (4.6)	137 (4.3)	−0.02	116 (3.7)	116 (3.7)	0.00
I63	6,373 (93.6)	3,014 (94.1)	0.02	2,970 (95)	2,970 (95)	0.00
CCI						
0	2,556 (37.5)	1,267 (39.6)	0.04	1,232 (39.4)	1,232 (39.4)	0.00
1	2,019 (29.7)	935 (29.2)	−0.01	920 (29.4)	920 (29.4)	0.00
2	1,187 (17.4)	542 (16.9)	−0.01	530 (17)	530 (17)	0.00
3	1,047 (15.4)	458 (14.3)	−0.03	445 (14.2)	445 (14.2)	0.00
Pre-stroke Polypharmacy	1,943 (28.5)	912 (28.5)	0.00	882 (28.2)	882 (28.2)	0.00
Underlying comorbidity						
Anxiety disorder	207 (3)	77 (2.4)	−0.04	101 (3.2)	76 (2.4)	−0.05
Cancer	385 (5.7)	190 (5.9)	0.01	164 (5.2)	185 (5.9)	0.03
Chronic back pain	3,194 (46.9)	605 (18.9)	−0.62	1,399 (44.7)	592 (18.9)	−0.58
Chronic liver failure	219 (3.2)	86 (2.7)	−0.03	94 (3)	84 (2.7)	−0.02
COPD	240 (3.5)	109 (3.4)	−0.01	131 (4.2)	108 (3.5)	−0.04
Cardiovascular disease	1,309 (19.2)	588 (18.4)	−0.02	588 (18.8)	577 (18.5)	−0.01
Dementia	376 (5.5)	328 (10.2)	0.18	178 (5.7)	314 (10)	0.16
Depressive disorder	273 (4)	88 (2.8)	−0.07	119 (3.8)	86 (2.8)	−0.06
Diabetes mellitus	2,063 (30.3)	889 (27.8)	−0.06	866 (27.7)	869 (27.8)	0.00
Hyperlipidemia	338 (5)	104 (3.3)	−0.09	146 (4.7)	102 (3.3)	−0.07
Hypertension	3,329 (48.9)	1,381 (43.1)	−0.12	1,556 (49.8)	1,347 (43.1)	−0.13
Osteoarthritis	2,354 (34.6)	599 (18.7)	−0.36	1,033 (33)	585 (18.7)	−0.33
Osteoporosis	472 (6.9)	109 (3.4)	−0.16	187 (6)	106 (3.4)	−0.12
Rheumatic arthritis	118 (1.7)	41 (1.3)	−0.04	39 (1.3)	37 (1.2)	−0.01
Renal failure	176 (2.6)	141 (4.4)	0.10	83 (2.7)	137 (4.4)	0.09
Schizophrenia	8 (0.1)	14 (0.4)	0.06	5 (0.2)	13 (0.4)	0.05

KM, korean medicine; COPD, chronic obstructive pulmonary disease; SMD, standardized mean difference.

The PS matching process within the polypharmacy group successfully balanced the covariates, with no significant differences between KM users (n = 3,127) and non-users (n = 3,127). However, disparities in the prevalence of chronic back pain and osteoarthritis persisted. Similarly, in the hyper-polypharmacy group, PS matching resulted in balanced covariates between KM users (n = 160) and non-users (n = 160), although differences in the incidence of chronic back pain remained.

### 3.2 Relationship of all-cause falls and mortality with KM use among patients with polypharmacy

Among patients in the polypharmacy group, those utilizing KM exhibited a higher risk of falls from all causes, with an adjusted hazard ratio (aHR) of 1.52 (95% confidence interval [CI] 1.325–1.751). Conversely, the all-cause mortality rate was lower in KM users compared with non-users, with an aHR of 0.66 (95% CI 0.59–0.74). These trends of increased falls and decreased mortality remained consistent across all univariate and multivariate models (Table 2).

**TABLE 2:** Risk of falls and deaths from all causes in polypharmacy, hyper-polypharmacy, and non-polypharmacy groups.

	Model 1 (Unadjusted)	Model 2	Model 3	Model 4	Model 5
	HR (95% CI)	aHR (95% CI)	aHR (95% CI)	aHR (95% CI)	aHR (95% CI)
Polypharmacy after stroke onset^a^					
Falls					
KM users (ref. non-users)	1.537(1.338–1.765)	1.525 (1.327–1.754)	1.525 (1.327–1.754)	1.522 (1.324–1.751)	1.523 (1.325–1.751)
Death					
KM users (ref. non-users)	0.760 (0.684–0.843)	0.735 (0.662–0.817)	0.728 (0.664–0.820)	0.683 (0.614–0.759)	0.663 (0.594–0.740)
Subgroup (Hyper-polypharmacy after stroke onset^b^)					
Falls					
KM users (ref. non-users)	1.08 (0.6–1.97)	1.01 (0.55–1.88)	1.01 (0.55–1.88)	0.99 (0.54–1.84)	0.99 (0.54–1.84)
Death					
KM users (ref. non-users)	0.65 (0.43–0.97)	0.64 (0.42–0.97)	0.64 (0.42–0.97)	0.64 (0.42–0.97)	0.64 (0.42–0.97)
Sensitivity analysis (Non-polypharmacy after stroke onset^c^)					
Falls					
KM users (ref. non-users)	1.08 (0.6–1.97)	1.01 (0.55–1.88)	1.01 (0.55–1.88)	0.99 (0.54–1.84)	0.99 (0.54–1.84)
Death					
KM users (ref. non-users)	0.65 (0.43–0.97)	0.64 (0.42–0.97)	0.64 (0.42–0.97)	0.64 (0.42–0.97)	0.64 (0.42–0.97)

^a^ Polypharmacy after stroke onset: defined as five or more medications prescribed for more than 270 days during the 1-year window after stroke onset.

^b^ Hyper-polypharmacy after stroke onset: defined as 10 or more medications prescribed for more than 270 days during the 1-year window after stroke onset.

^c^ Non-polypharmacy after stroke onset: defined as fewer than five medications prescribed for more than 270 days during the 1-year window after stroke onset.

Model 1, univariate; Model 2, adjusted for age group, sex, and economic status; Model 3, adjusted for age group, sex, economic status, and types of stroke; Model 4, adjusted for age group, sex, economic status, types of stroke, and CCI; Model 5, adjusted for age group, sex, economic status, types of stroke, CCI, and pre-stroke polypharmacy (defined as five or more medications prescribed for more than 180 days during the 1-year window before stroke onset).

KM, korean medicine; aHR, adjusted hazard ratio; CI, confidence interval.

### 3.3 Patient characteristics of the hyper-polypharmacy cohort

Compared with the PS-matched polypharmacy cohort, which showed an even distribution across age groups, the hyper-polypharmacy group had a higher concentration of patients aged 70–79 and an underrepresentation of those above 85. The hyper-polypharmacy group also had higher CCI scores, with 67.5% reporting pre-stroke polypharmacy, compared with only 28.2% in the general polypharmacy group. This group exhibited an overall increase in most prespecified comorbidities, including anxiety disorder, cancer, chronic liver failure, chronic obstructive pulmonary disease, cardiovascular disease, dementia, depressive disorder, diabetes mellitus, hyperlipidemia, rheumatoid arthritis, renal failure, and schizophrenia. In contrast, KM users in the hyper-polypharmacy group showed a decreased prevalence of chronic back pain, hypertension, osteoarthritis, and osteoporosis (Supplementary Table S4).

### 3.4 Relationship of all-cause falls and mortality with KM use among patients with hyper-polypharmacy

In the hyper-polypharmacy group, the risk of falls from all causes was not significantly different between KM users and non-users (aHR 0.99, 95% CI 0.54–1.84). However, KM users had a lower all-cause mortality rate (aHR 0.64, 95% CI 0.42–0.97). These findings of no significant difference in falls and a decreased mortality risk were consistent across all univariate and multivariate models (Table 2).

### 3.5 Sensitivity analyses

No notable differences in the risk of falls and deaths from all causes were observed in the subgroup and sensitivity analyses. For the PS-matched non-polypharmacy group (Supplementary Table S5), the risk of falls was not statistically different between KM users and non-users (aHR 0.99, 95% CI 0.54–1.84). On the contrary, the decreased mortality with KM use remained significant (aHR 0.64, 95% CI 0.42–0.97, Table 2).

When prespecified comorbidities were included as covariates (Supplementary Table S6), the trends of increased falls (aHR 1.45, 95% CI 1.25–1.69) and decreased mortality (aHR 0.72, 95% CI 0.65–0.81) in the polypharmacy group due to KM use were robust. In the PS-matched hyper-polypharmacy subgroup, fall risk remained unaffected by the inclusion of prespecified comorbidities as covariates (aHR 1.11, 95% CI 0.56–2.19). Similarly, the reduced all-cause mortality in KM users remained insignificant when prespecified comorbidities were considered (aHR 0.73, 95% CI 0.47–1.14). In the PS-matched non-polypharmacy group, fall risk increased by the inclusion of prespecified comorbidities (aHR 1.22, 95% CI 0.74–1.39). The reduced all-cause mortality remained insignificant (aHR 0.79, 95% CI 0.72–0.88).

When medication counts were included as covariates (Supplementary Table S7), the trends of increased falls (aHR 1.45, 95% CI 1.25–1.69) and decreased mortality (aHR 0.72, 95% CI 0.67–0.77) in the polypharmacy group owing to KM use were consistent. In the PS-matched hyper-polypharmacy subgroup, fall risk remained unaffected by the inclusion of medication counts as covariates (aHR 1.12, 95% CI 0.56–2.24). Similarly, the reduced all-cause mortality in KM users remained insignificant when medication counts were considered (aHR 0.72, 95% CI 0.46–1.14). In the PS-matched non-polypharmacy group, fall risk increased by the inclusion of medication counts (aHR 1.23, 95% CI 1.08–1.40). The reduced all-cause mortality remained insignificant (aHR 0.77, 95% CI 0.70–0.85).

The potential interaction between KM use and medication counts (Supplementary Table S8) for fall risk was not statistically significant (p = 0.844), indicating that the effect of KM on the hazard of falls does not significantly vary with the number of medications. The hazard ratios for KM use showed consistent results for falls up to 10 medications, with a nonsignificant trend for 10 or more medications. Similarly, the interaction between KM use and medication counts for all-cause mortality was not significant (p = 0.526). This suggests that the effect of KM on the risk of death does not significantly differ based on medication counts, with a consistent decrease in mortality risk for KM users compared with non-users across various levels of medication counts.

Lastly, incidence-rate ratios derived from the unmatched cohort paralleled those observed after propensity matching (Supplementary Table S9), supporting the robustness of the primary findings. We then used the same crude event counts to bound the influence of mortality as a competing risk for falls. Under a maximal-overlap scenario—every patient who fractured eventually died within the follow-up window—deaths still represented 16% of all first events. Under a no-overlap scenario—no faller died during follow-up—deaths rose to 54% of first events. Because even the most conservative bound exceeds the commonly cited 15% threshold at which cause-specific and Fine–Gray estimates begin to diverge, competing mortality is likely to meaningfully affect fracture estimates. A person-time–based calculation yielded a concordant result: assuming constant hazards, the probability that death occurs before a first fracture was approximately 52%. These figures confirm that mortality is a substantial competing event and may partly underlie the higher observed fall rates in KM users.

## 4 Discussion

We investigated how KM usage—primarily acupuncture—affects falls and mortality among older stroke survivors with varying degrees of polypharmacy. Using the NHIS database, we analyzed 25,034 patients who experienced a stroke, categorized them into polypharmacy (≥5 medications) or hyper-polypharmacy (≥10 medications) groups, and examined two outcomes over 3 years: all-cause mortality and falls resulting in fractures. PS matching was used to minimize baseline differences between KM users and non-users, balancing factors such as demographics, comorbidities, and stroke severity. We then applied Cox proportional hazards models to assess how KM influenced our outcomes, performing sensitivity checks to confirm the robustness of the results.

In the polypharmacy group, KM use was associated with a heightened risk of falls (aHR 1.52) but a reduced risk of all-cause mortality (aHR 0.66) compared with non-use. This seemingly paradoxical pattern of higher fall incidence yet lower all-cause mortality among KM users can be reconciled by mechanisms well documented in stroke-rehabilitation research, even though functional status could not be measured directly in the NHIS data. Multiple studies including randomized controlled trials show that post-stroke acupuncture, combined with targeted physical training and herbal prescriptions, accelerate gains in motor strength, gait speed, and activities of daily living, and shorten the modified Rankin Scale recovery (Liu et al., 2009; Yan and Hui-Chan, 2009; Zhao et al., 2009; Hong et al., 2024; Hu et al., 2024). Improved mobility enlarges patients’ “activity envelope”; because most post-stroke falls occur during routine ambulation rather than high-risk tasks (Wang et al., 2025; Morone et al., 2014), increased activity envelope predictably yields into more opportunities to fall. At the same time, enhanced locomotion curtails secondary complications such as pneumonia, pressure ulcers, sarcopenia and facilitates cardiovascular conditioning, which together have been linked to better long-term survival (Lui and Nguyen, 2018), which inadvertently raises fall risk if patients do not receive adequate balance training (Morone et al., 2014; Wei et al., 2019).

In contrast, among those with hyper-polypharmacy, KM did not significantly change fall risk (aHR 0.99) but still lowered all-cause mortality (aHR 0.64). As patients with severe polypharmacy often have more advanced multimorbidity and frailty, KM’s positive impact on mobility might be less pronounced in that group, explaining the nonsignificant difference in falls. These findings were consistent across various models and sensitivity analyses, indicating a robust protective effect of KM on mortality regardless of medication burden. The absence of significant interaction effects between KM use and medication counts for both falls and mortality suggests that the benefits of KM, particularly in reducing mortality, are broadly applicable across medication complexity levels.

Although the primary KM intervention was acupuncture, another major therapy is herbal medicine. Owing to gaps in insurance coverage, herbal medicine use is not fully captured in the NHIS claims data. However, nationwide survey data indicate that a substantial portion of KM users also utilize herbal remedies: 94.3% use acupuncture, 28.5% use herbal extracts, and 26.7% use herbal decoctions (Development, 2024). This widespread use likely means most herbal medicine users were included in our KM cohort. By analyzing how an insured medical services provided within the scope of KM interacts with extensive Western polypharmacy, our study tackles a genuinely ethnopharmacological question: can a traditional, system-based medical paradigm mitigate drug-burden complications in real-world stroke care? Herbal medicine has been reported to alleviate neurologic deficits (Yang et al., 2016; Yu et al., 2015; Han et al., 2018) and improve independence (Goto et al., 2011). By enhancing motor function and mobility, herbal therapies could further increase the risk of falls—walking is the most common activity preceding falls in stroke survivors (Weerdesteyn et al., 2008)—but also contribute to better overall recovery. Although the NHIS database does not allow itemization of separate herbs and herbal decoctions, their silent presence implies the integrated KM practice provided to the treatment group actually seen in clinics in real-world settings.

Well-documented herbal effects that we could not model quantitatively nevertheless remain clinically relevant for post-stroke patients. The reduction in mortality may be attributed to KM’s ability to improve various factors that can worsen prognosis in the post-stroke recovery period in addition to functional recovery, such as acupuncture improving post-stroke dysphagia (Long and Wu, 2012; Cohen et al., 2016), herbal medicines inhibiting the progression of acute cerebral infarction (Jung et al., 2003), preventing aspiration pneumonia (Iwasaki et al., 2007), promoting negative conversion of resistant bacteria (Kohno et al., 2021), reducing early neurological deterioration (Tian et al., 2021), and preventing recurrence (Lee et al., 2023; Jung et al., 2018). Collectively these mechanisms—neuroprotection, infection control, and secondary prevention—could explain the lower all-cause mortality we observed among KM users. In patients with severe multimorbidity, however, KM’s impact on mobility could be blunted (Yao et al., 2020) owing to increased medications (Muth and Glasziou, 2015), potentially explaining why fall risk was unchanged in the hyper-polypharmacy group. Even though individual herbal prescriptions were unmeasurable, the aforementioned literature shows that they plausibly augment the neuromuscular and systemic benefits of acupuncture while also carrying their own risk-benefit trade-offs. Recognizing those unseen contributions strengthens the biological coherence of our findings and highlights why future linked-dataset or prospective studies that capture uninsured herbal dispensing are essential for a full pharmacological accounting of KM in stroke rehabilitation.

While the neuromotor benefits of acupuncture and other KM modalities are well documented, the higher fall rate observed in KM recipients is more plausibly driven by survivor bias than by treatment-related harm. Our competing-risk sensitivity analysis showed that deaths accounted for 16%–54% of all first events and that, assuming constant hazards, the probability of death preceding a first fracture was ≈52%. Because KM users experienced lower early mortality, they contributed more person-time at risk and therefore had more opportunity to incur a fall. When this prolonged exposure is combined with the greater mobility that KM appears to foster, the crude fall incidence is inevitably inflated without implying an intrinsic increase in fall propensity. Accordingly, the excess falls should be viewed not as an adverse effect of KM itself but as the by-product of improved survival and functional recovery. Future studies with patient-level event ordering and richer clinical detail are needed to apply formal competing-risk methods—such as Fine–Gray sub-distribution models—and to verify this interpretation.

In the matched cohort, KM users had a higher prevalence of chronic pain conditions such as low back pain and osteoarthritis, suggesting that individuals with persistent pain may be more inclined to seek KM owing to its perceived or actual effectiveness. Acupuncture can alleviate chronic pain and encourage greater activity (Vickers et al., 2018), potentially improving quality of life (Manyanga et al., 2014; Seca et al., 2019). The resulting improvement in pain control and quality of life may encourage increased activity, contributing to fall risk but also enhancing overall health. This increased activity, however, can raise fall risk if patients resume normal routines without proper balance training or physical conditioning (Skelton, 2023). Nonetheless, better pain management may also slow the progression of other chronic conditions, potentially contributing to improved survival (Nielsen et al., 2022). While these benefits do not inherently lower fall risk, they highlight acupuncture’s broader role in chronic disease management, which can enhance patients’ overall longevity despite an accompanying increase in activity-related hazards.

Our study offers a fresh perspective on managing polypharmacy in older stroke survivors. Established criteria (Beers (Panel, 2015), STOPP/START (O'Mahony et al., 2023)) aim to reduce inappropriate prescribing but have shown mixed results in preventing adverse events (Frankenthal et al., 2014; Cole et al., 2023). In South Korea, where prescribing practices differ from Western settings (Lee et al., 2022), KM could serve as an integrative approach, potentially mitigating some negative outcomes of polypharmacy without reducing medication counts directly. We could not confirm whether KM helps discontinue medications, as we focused only on whether participants were in a polypharmacy state. Nevertheless, our findings strongly suggest that KM use is associated with improved survival regardless of medication load.

This study has limitations. First, we did not assess whether the number of medications changed over time or whether fewer drugs, in conjunction with KM, led to improved outcomes. Second, while acupuncture (including electroacupuncture) formed the main exposure, other integrative therapies such as herbal medicine might also have contributed to the outcomes (Lee et al., 2024). Third, the findings are specific to older stroke survivors and may not translate to other populations. We also did not examine prognostic factors such as cognitive impairment or frailty, which could influence falls and mortality.

This study has several strengths and limitations that must be weighed when interpreting the findings. On the strength side, we applied rigorous propensity-score methods and a large, nationally representative cohort of older stroke survivors, enabling precise estimates for outcomes such as post-stroke mortality. Nevertheless, limitations remain. First, we could not assess the relationship between the changes of the number of medications and the outcomes, precluding evaluation of whether subsequent de-prescribing—or the synergy between fewer drugs and Korean Medicine (KM)—mediated the observed associations. Second, although acupuncture (including electroacupuncture) constituted the primary exposure, ancillary KM modalities such as herbal prescriptions and manual therapies were not covered by NHIS and therefore were incompletely recorded in the database. It is important to note that these modalities may have contributed to both fall risk and survival. Third, several prognostic factors unavailable in NHIS database—including neurological severity (e.g., NIHSS), rehabilitation dose, family support, cognitive impairment, and frailty—could confound the results; if KM users were functionally milder or enjoyed greater social resources, the mortality benefit could be over-estimated and the excess falls under-estimated, whereas preferential KM use by more disabled patients would bias effects toward the null. Fourth, we could not evaluate pharmacodynamic interactions involving high-risk combinations—e.g., anticoagulants taken concurrently with selective-serotonin reuptake inhibitors or benzodiazepines—even though these pairings are well known to increase bleeding, sedation, and fall propensity. Reliable identification of such interactions would require granular data on dose, formulation, and administration timing, none of which are available in the NHIS claims files; thus a clinically meaningful co-exposure definition could not be constructed for the present analysis. Finally, because the cohort comprised elderly stroke survivors, generalizability to younger patients or to non-stroke populations is uncertain. Prospective studies that track dynamic medication exposure and incorporate detailed functional, cognitive, and social metrics are warranted to validate and extend these findings.

## 5 Conclusion

Among older stroke survivors, initiation of Korean Medicine (KM) within the first post-stroke year was linked to a higher incidence of falls yet a lower 3-year all-cause mortality, and these associations persisted regardless of baseline polypharmacy status. In the hyper-polypharmacy subgroup, KM left fall risk unchanged but still conferred a survival advantage. The absence of significant interaction terms indicates that KM neither amplifies nor attenuates the effect of polypharmacy on either outcome; rather, its mortality benefit appears broadly applicable, whereas the excess falls likely reflect enhanced mobility and survivor bias. Prospective studies with granular medication data and detailed KM exposure—including uninsured herbal prescriptions—are warranted to determine whether specific KM components can simultaneously reduce drug burden and adverse clinical events in diverse geriatric populations.

## Data Availability

The raw data supporting the conclusions of this article will be made available by the authors, without undue reservation.
