# Supplementary material for: Association of Korean Medicine and polypharmacy with fall risk and mortality in older adults with stroke
Source: Front Pharmacol. 2025 Jun 19;16:1621819. doi: 10.3389/fphar.2025.1621819 (PMC12222143; doi:10.3389/fphar.2025.1621819)
Supplement: Supplementary file 1 [file Table1.docx]

**Supplementary table 1.**

| **Categories** | **Full names of medications** |
| --- | --- |
| ANTITHROMBOTIC AGENTS | aspirin(enteric coated) (as aspirin), clopidogrel bisulfate (as clopidogrel), warfarin sodium, cilostazol, triflusal, clopidogrel resinate (as clopidogrel), aspirin, beraprost sodium, aspirin(encapsulated), clopidogrel besylate (as clopidogrel), dabigatran etexilate mesylate (as dabigatran etexilate), limaprost α-cyclodextrin (as limaprost), pentoxifylline, rivaroxaban(micronized), sarpogrelate hydrochloride |
| CARDIOVASCULAR AGENTS | amlodipine besylate (as amlodipine), hydrochlorothiazide, losartan potassium (as losartan), telmisartan, valsartan, amlodipine camsylate (as amlodipine), olmesartan medoxomil, fimasartan potassium trihydrate (as fimasartan potassium), candesartan cilexetil, irbesartan, cilnidipine, S-amlodipine nicotinate (as S-amlodipine), amlodipine maleate (as amlodipine), amlodipine orotate (as amlodipine), benidipine hydrochloride, lercanidipine hydrochloride, nifedipine, olmesartan medoxomi, perindopril tertbutylamine, S-amlodipine besylate (as S-amlodipine), S-amlodipine gentisate (as S-amlodipine), amlodipine nicotinate (as amlodipine), lacidipine, lisinopril, ramipril, bisoprolol fumarate, carvedilol, atenolol, spironolactone, furosemide, nicorandil, digoxin, isosorbide mononitrate, trimetazidine hydrochloride, chlorthalidone, desmopressin acetate (as desmopressin), mesoglycan sodium, nebivolol hydrochloride (as nebivolol), propranolol hydrochloride, S-atenolol, amiodarone hydrochloride, diltiazem hydrochloride, isosorbide dinitrate, torasemide, cytochrome C |
| ANTIDIABETIC AGENTS | metformin hydrochloride, glimepiride, gliclazide, sitagliptin phosphate hydrate (as sitagliptin), voglibose, pioglitazone hydrochloride (as pioglitazone), vildagliptin, thioctic acid(α-lipoic acid), glibenclamide, linagliptin, bilberry fruit dried ext. |
| LIPID MODIFYING AGENTS | atorvastatin calcium (as atorvastatin), simvastatin, pitavastatin calcium, rosuvastatin calcium (as rosuvastatin), ezetimibe, fenofibrate, pravastatin sodium, omega-3-acid ethyl esters 90, fenofibrate granule(micronized) (as fenofibrate) |
| NON-NARCOTIC ANALGESICS AND ANTIEPILEPTICS | tramadol hydrochloride, aceclofenac, gabapentin, meloxicam, acetaminophen, sodium valproate, celecoxib, levetiracetam, pregabalin, afloqualone, carbamazepine, topiramate, other antiinflammatory and antirheumatic agents (non-steroids), acetaminophen(encapsulated), aescin, lamotrigine, orphenadrine hydrochloride, oxcarbazepine , talniflumate, thiocolchicoside, eperisone hydrochloride |
| ANTI-PARKINSON AND ANTI-DEMENTIA AGENTS | choline alfoscerate, acetyl L-carnitine hydrochloride (as acetyl L-carnitine), nicergoline, donepezil hydrochloride, ginkgo biloba leaf ext., oxiracetam, folic acid, levodopa, galantamine hydrobromide (as galantamine), memantine hydrochloride (as memantine), rivastigmine tartrate (as rivastigmine), benserazide hydrochloride (as benserazide), ropinirole hydrochloride (as ropinirole), amantadine sulfate, carbidopa hydrate (as carbidopa), carbidopa monohydrate (as carbidopa), entacapone, trihexyphenidyl hydrochloride |
| PSYCHOLEPTICS AND HYPNOTICS | quetiapine fumarate (as quetiapine), amitriptyline hydrochloride, clonazepam, diazepam, escitalopram oxalate (as escitalopram), lorazepam, triazolam, alprazolam, citalopram hydrobromide (as citalopram), etizolam, haloperidol, risperidone, sodium tianeptine, zolpidem tartrate |
| GASTROINTESTINAL AGENTS | rebamipide, ranitidine hydrochloride (as ranitidine), artemisiae argyi folium 95% ethanol ext.(20→1), sucralfate hydrate (as sucrose octasulfate), tripotassium bismuth dicitrate 0.1g(as bismuth oxide 40.5mg, as potassium 12mg), magnesium hydroxide, dimenhydrinate, mosapride citrate hydrate (as mosapride), almagate, itopride hydrochloride, lafutidine, levosulpiride, rabeprazole sodium, trimebutine maleate, corydalis tuber·pharbitis seed(5:1) 50% ethanol ext.(9.5~11.5→1), dehydrocholic acid, lactulose cone., magnesium oxide, nizatidine, pancreatin, simethicone, calcium carbonate, calcium carbonate (as calcium), calcium polycarbophil, benexate betadex, biphenyl dimethyl dicarboxylate, bupropion hydrochloride, cimetidine, diomagnite, domperidone maleate (as domperidone), ecabet sodium, esomeprazol magnesium (as esomeprazol), famotidine, lansoprazole, magnesium trihydrate salt of chenodesoxycholic acid and ursodesoxycholic acid, megestrol acetate, metoclopramide, octylonium bromide, omeprazole granule(enteric coated) (as omeprazole), ornithine aspartate, teprenone, ursodeoxycholic acid, calcium carbonate(precipitated) |
| UROLOGICAL AGENTS | tamsulosin hydrochloride, dutasteride, finasteride, doxazosin mesylate (as doxazocin), propiverine hydrochloride, silodosin, bethanechol chloride, terazosin hydrochloride (as terazosin),trospium chloride |
| RESPIRATORY AGENTS | erdosteine, acetylcysteine, chlorpheniramine maleate, theophylline, bromhexine hydrochloride, doxofylline, levodropropizine, streptokinase·streptodornase(streptokinase 10KI.U,streptodornase 2.5KI.U), ambroxol hydrochloride, ammonium chloride, dried coptidis rhizoma ext. (4.5~7:1), dried ivy leaf ext. (5~7.5:1), guaifenesin, procaterol hydrochloride, tiotropium bromide monohydrate(micronized) (as tiotropium) |
| OTHER AGENTS | cholecalciferol, bromelain, dihydrocodeine tartrate, ferrous sulfate dried (as Fe II), levothyroxine sodium, mecobalamin, DL-methylephedrine hydrochloride, allopurinol, bepotastine besilate, calcitriol, calcium citrate (as calcium), calcium gluconate hydrate, calcium lactate hydrate, cholecalciferol (vitamin D3), chondroitin sodium sulfate, ergocalciferol, garlic oil, hydroxyzine hydrochloride, ketotifen fumarate (as ketotifen), methylephedrine HCl, polystyrene sulfonate calcium, potassium chloride, prednisolone, pyridoxine hydrochloride, sodium alendronate (as alendronic acid), tibolone, tosufloxacin tosylate |

**Supplementary table 2.**

| **ICD-10 codes** | | **Disease** |
| --- | --- | --- |
| M codes | M48.3 | Traumatic spondylopathy |
|  | M48.4 | Fatigue fracture of vertebra |
|  | M48.5 | Collapsed vertebra |
|  | M80 | Osteoporosis with pathological fracture |
|  | M84.3 | Stress fracture |
| S codes | S02.0 | Fracture of vault of skull |
|  | S02.1 | Fracture of base of skull |
|  | S22 | Fracture of ribs, sternum and thoracic spine |
|  | S23 | Dislocation, sprain and strain of joints and ligaments of thorax |
|  | S32 | Fracture of lumbar spine and pelvis |
|  | S33 | Dislocation, sprain and strain of joints and ligaments of lumbar spine and pelvis |
|  | S72 | Fracture of femur |
|  | S73 | Dislocation, sprain and strain of joint and ligaments of hip |

**Supplementary table 3.**

| **Diagnosis** | **ICD-10-CM code and definition** | **Diagnostic definition** |
| --- | --- | --- |
| **Inclusion criteria** |  |  |
| Cerebrovascular diseases | I60, I61, I63 | initial diagnosis in 1/1/2015 - 12/31/2015 |
| **Exclusion criteria** |  |  |
| Cerebrovascular diseases (all types) | I60, I61, I62, I63, I64, I65, I66, I67, I68, I69 | history of cerebrovascular diagnosis since 1/1/2002 to initial diagnosis |
| **Exposure** |  |  |
| Polypharmacy (after stroke) |  | Polypharmacy of routine stroke prescriptions (≥5 prescriptions with ≥270 days of supplies in one-year period since the initial stroke diagnosis in 2015) |
|  |  | *Sensitivity analyses were conducted using the following definions: 1) ≥5 prescriptions with ≥180 days of supplies, and 2) ≥10 prescriptions with ≥270 days of supplies. |
| **Covariates** |  | |
| **Polypharmacy (before stroke)** |  | Polypharmacy of routine stroke prescriptions **prior to** the initial stroke diagnosis in 2015 |
| **Comorbid conditions within one-year period before the initial stroke diagnosis** |  | |
| Diabetes mellitus (both with and without complications) | E10, E11, E12, E13, E14 | |
| Cancer (all types) | C00, C01, C02, C03, C04, C05, C06, C07, C08, C09, C10, C11, C12, C13, C14, C15, C16, C17, C18, C19, C20, C21, C22, C23, C24, C25, C26, C30, C31, C32, C33, C34, C37, C38, C39, C40, C41, C43, C45, C46, C47, C48, C49, C50, C51, C52, C53, C54, C55, C56, C57, C58, C60, C61, C62, C63, C64, C65, C66, C67, C68, C69, C70, C71, C72, C73, C74, C75, C76, C77, C78, C79, C80, C81, C82, C83, C84, C85, C88, C90, C91, C92, C93, C94, C95, C96, C97 | |
| Cancer (in situ) | D00, D01, D02, D03, D04, D05, D06, D07, D08, D09 | |
| Chronic back pain | M51, M53, M54 | |
| Osteoarthritis | M15, M16, M17, M18, M19 | |
| Rheumatoid | M05, M06 | |
| Osteoporosis | M80, M81, M82 | |
| Chronic obstructive pulmonary disease (COPD) | J43, J44 | |
| Dementia | F00, F01, F02, F03, G30 | |
| Schizophrenia | F20, F21 | |
| Depressive disorders | F32, F33 | |
| Anxiety disorders | F40, F41 | |
| Hyperlipidemia | E78 | |
| Hypertension | I10, I11, I12, I13, I15 | |
| Cardiovascular diseases | I05, I06, I07, I08, I09, I20, I21, I22, I23, I24, I25, I26, I27, I30, I31, I32, I33, I34, I35, I36, I37, I38, I39, I40, I41, I42, I43, I44, I45, I46, I47, I48, I49, I50, I51, I52 | |
| Renal failure | N17, N18, N19 | |
| Chronic liver diseases | K72, K73, K74, K75, K76, K77 | |
| **Charlson Comorbidity Index (Score)** |  | |
| Myocardial infarction | I21, I22, I252 | |
| Congestive heart failure | I099, I110, I130, I132, I255, I420, I425, I426, I427, I428, I429, I43, I50, P290 | |
| Peripheral vascular disease | I70, I71, I731, I738, I739, I771, I790, I792, K551, K558, K559, Z958, Z959 | |
| Cerebrovascular disease | G45, G46, I60, I61, I62, I63, I64, I65, I66, I67, I68, I69, H340 | |
| Dementia | F00, F01, F02, F03, G30, F051, G311 | |
| Chronic pulmonary disease | I278, I279, J40, J41, J42, J43, J44, J45, J46, J47, J60, J61, J62, J63, J64, J65, J66, J67, J684, J701, J703 | |
| Connective tissue disease | M05, M06, M315, M32, M33, M34, M351, M353, M360 | |
| (Rheumatologic disease) |  |  |
| Peptic ulcer disease | K25, K26, K27, K28 | |
| Mild liver disease | B18, K700, K701, K702, K703, K709, K713, K714, K715, K717, K73, K74, K760, K762, K763, K764, K768, K769, Z944 | |
| Moderate or severe liver disease (3) | I850, I859, I864, I982, K704, K711, K721, K729, K765, K766, K767 | |
| Diabetes without complications | E100, E101, E106, E108, E109, E110, E111, E116, E118, E119, E120, E121, E126, E128, E129, E130, E131, E136, E138, E139, E140, E141, E146, E148, E149 | |
| Diabetes with complications (2) | E102, E103, E104, E105, E107, E112, E113, E114, E115, E117, E122, E123, E124, E125, E127, E132, E133, E134, E135, E137, E142, E143, E144, E145, E147 | |
| Paraplegia and hemiplegia (2) | G041, G114, G800, G801,G802, G81, G82, G830, G831, G832, G833, G834, G839 | |
| Renal disease (2) | I120, I131, N030, N031, N032, N033, N034, N035, N036, N037, N038, N039, N050, N051, N052, N053, N054, N055, N056, N057, N058, N059, N18, N19, N250, Z490, Z491, Z492, Z940, Z992 | |
| Cancer (2) | C00, C01, C02, C03, C04, C05, C06, C07, C08, C09, C10, C11, C12, C13, C14, C15, C16, C17, C18, C19, C20, C21, C22, C23, C24, C25, C26, C30, C31, C32, C33, C34, C37, C38, C39, C40, C41, C43, C45, C46, C47, C48, C49, C50, C51, C52, C53, C54, C55, C56, C57, C58, C60, C61, C62, C63, C64, C65, C66, C67, C68, C69, C70, C71, C72, C73, C74, C75, C76, C81, C82, C83, C84, C85, C88, C90, C91, C92, C93, C94, C95, C96, C97 | |
| Metastatic carcinoma (6) | C77, C78, C79, C80 | |
| AIDS/HIV (6) | B20, B21, B22, B24 | |
| **Demographic variables** |  |  |
| Age group |  | 65-74, 75-84, 85 or older |
| Sex |  |  |
| Income | Low (Level 0) | Medicaid |
|  | Lower-middle (Level 1-5) | NHIS |
|  | Low (Level 6-10) |  |
|  | Middle (Level 11-15) |  |
|  | High (Level 16-20) |  |
| **Outcome** |  |  |
| Falls | M48.3, M48.4, M48.5, M80, M84.3, S02.0, S02.1, S22, S23, S32, S33, S72, S73 | |
| All-cause mortality |  | Followed up for 3 years since the index date |

**Supplementary Table 4. Baseline characteristics of Korean Medicine treatment (KM) users and non-users among stroke patients with hyper-polypharmacy.**

|  | **After propensity score matching (n = 320)** | | |
| --- | --- | --- | --- |
|  | **KM users (n = 160)** | **Non-users (n = 160)** | **SMD** |
| **Sex** |  |  |  |
| **Male** | 92 (57.5) | 92 (57.5) | 0.00 |
| **Female** | 68 (42.5) | 68 (42.5) | 0.00 |
| **Age groups** |  |  |  |
| **65-69** | 39 (24.4) | 39 (24.4) | 0.00 |
| **70-74** | 34 (21.3) | 34 (21.3) | 0.00 |
| **75-79** | 49 (30.6) | 49 (30.6) | 0.00 |
| **80-84** | 30 (18.8) | 30 (18.8) | 0.00 |
| **85-89** | 7 (4.4) | 7 (4.4) | 0.00 |
| **90-** | 1 (0.6) | 1 (0.6) | 0.00 |
| **Area** |  |  |  |
| **Metropolitan** | 92 (57.5) | 80 (50) | 0.15 |
| **Rural** | 68 (42.5) | 80 (50) | -0.15 |
| **Economic status** |  |  |  |
| **Low** | 20 (12.5) | 12 (7.5) | 0.17 |
| **Lower middle** | 16 (10) | 21 (13.1) | -0.10 |
| **Middle** | 31 (19.4) | 23 (14.4) | 0.13 |
| **Upper middle** | 34 (21.3) | 32 (20) | 0.03 |
| **High** | 54 (33.8) | 70 (43.8) | -0.21 |
| **Unknown** | 5 (3.1) | 2 (1.3) | 0.13 |
| **Types of stroke** |  |  |  |
| **I60** | - | - | 0.00 |
| **I61** | 3 (1.9) | 3 (1.9) | 0.00 |
| **I63** | 157 (98.1) | 157 (98.1) | 0.00 |
| **CCI_GROUP** |  |  |  |
| **0** | 27 (16.9) | 27 (16.9) | 0.00 |
| **1** | 38 (23.8) | 38 (23.8) | 0.00 |
| **2** | 45 (28.1) | 45 (28.1) | 0.00 |
| **3** | 50 (31.3) | 50 (31.3) | 0.00 |
| **Pre-stroke Polypharmacy** | 108 (67.5) | 108 (67.5) | 0.00 |
| **Underlying comorbidity** |  |  |  |
| **Anxiety disorder** | 10 (6.3) | 8 (5) | 0.05 |
| **Cancer** | 10 (6.3) | 16 (10) | -0.14 |
| **Chronic back pain** | 43 (26.9) | 83 (51.9) | -0.53 |
| **Chronic liver failure** | 8 (5) | 5 (3.1) | 0.09 |
| **COPD** | 7 (4.4) | 12 (7.5) | -0.13 |
| **Cardiovascular disease** | 51 (31.9) | 56 (35) | -0.07 |
| **Dementia** | 22 (13.8) | 17 (10.6) | 0.10 |
| **Depressive disorder** | 13 (8.1) | 8 (5) | 0.13 |
| **Diabetes mellitus** | 85 (53.1) | 83 (51.9) | 0.03 |
| **Hyperlipidemia** | 6 (3.8) | 6 (3.8) | 0.00 |
| **Hypertension** | 62 (38.8) | 71 (44.4) | -0.11 |
| **Osteoarthritis** | 37 (23.1) | 49 (30.6) | -0.17 |
| **Osteoporosis** | 6 (3.8) | 8 (5) | -0.06 |
| **Rheumatic arthritis** | 5 (3.1) | 7 (4.4) | -0.07 |
| **Renal failure** | 18 (11.3) | 21 (13.1) | -0.06 |
| **Schizophrenia** | 2 (1.3) | - | 0.16 |

**Supplementary Table 5. Baseline characteristics of Korean Medicine treatment (KM) users and non-users among stroke patients with non-polypharmacy.**

|  | **Before propensity score matching (n = 15,023)** | | | **After propensity score matching (n = 8,888)** | | |
| --- | --- | --- | --- | --- | --- | --- |
|  | **KM users**  **(n = 10,506)** | **Non-users**  **(n = 4,517)** | **SMD** | **KM users**  **(n = 4,444)** | **Non-users**  **(n = 4,444)** | **SMD** |
| **Sex** |  |  |  |  |  |  |
| **Male** | 4,613 (43.9) | 2,640 (58.5) | 0.29 | 2,551 (57.4) | 2,504 (56.4) | -0.02 |
| **Female** | 5,893 (56.1) | 1,877 (41.6) | -0.29 | 1,893 (42.6) | 1,940 (43.7) | 0.02 |
| **Age groups** |  |  |  |  |  |  |
| **65-69** | 2,714 (25.8) | 1,104 (24.4) | -0.03 | 1,101 (24.8) | 1,101 (24.8) | 0.00 |
| **70-74** | 2,961 (28.2) | 1,033 (22.9) | -0.12 | 1,032 (23.2) | 1,032 (23.2) | 0.00 |
| **75-79** | 2,524 (24) | 1,041 (23.1) | -0.02 | 1,040 (23.4) | 1,040 (23.4) | 0.00 |
| **80-84** | 1,555 (14.8) | 731 (16.2) | 0.04 | 728 (16.4) | 728 (16.4) | 0.00 |
| **85-89** | 599 (5.7) | 409 (9.1) | 0.13 | 401 (9) | 401 (9) | 0.00 |
| **90-** | 153 (1.5) | 199 (4.4) | 0.18 | 142 (3.2) | 142 (3.2) | 0.00 |
| **Area** |  |  |  |  |  |  |
| **Metropolitan** | 5,985 (57) | 2,545 (56.3) | -0.01 | 2,551 (57.4) | 2,504 (56.4) | -0.02 |
| **Rural** | 4,521 (43) | 1,972 (43.7) | 0.01 | 1,893 (42.6) | 1,940 (43.7) | 0.02 |
| **Economic status** |  |  |  |  |  |  |
| **Low** | 606 (5.8) | 341 (7.6) | 0.07 | 206 (4.6) | 329 (7.4) | 0.12 |
| **Lower middle** | 1,754 (16.7) | 741 (16.4) | -0.01 | 720 (16.2) | 730 (16.4) | 0.01 |
| **Middle** | 1,381 (13.1) | 634 (14) | 0.03 | 627 (14.1) | 625 (14.1) | 0.00 |
| **Upper middle** | 2,168 (20.6) | 928 (20.5) | 0.00 | 888 (20) | 917 (20.6) | 0.02 |
| **High** | 4,404 (41.9) | 1,817 (40.2) | -0.03 | 1,917 (43.1) | 1,788 (40.2) | -0.06 |
| **Unknown** | 193 (1.8) | 56 (1.2) | -0.05 |  |  |  |
| **Types of stroke** |  |  |  |  |  |  |
| **I60** | 360 (3.4) | 147 (3.3) | -0.01 | 138 (3.1) | 138 (3.1) | 0.00 |
| **I61** | 826 (7.9) | 322 (7.1) | -0.03 | 304 (6.8) | 304 (6.8) | 0.00 |
| **I63** | 9,320 (88.7) | 4,048 (89.6) | 0.03 | 4,002 (90.1) | 4,002 (90.1) | 0.00 |
| **CCI_GROUP** |  |  |  |  |  |  |
| **0** | 4,998 (47.6) | 2,346 (51.9) | 0.09 | 2,321 (52.2) | 2,321 (52.2) | 0.00 |
| **1** | 3,165 (30.1) | 1,288 (28.5) | -0.04 | 1,265 (28.5) | 1,265 (28.5) | 0.00 |
| **2** | 1,404 (13.4) | 524 (11.6) | -0.05 | 513 (11.5) | 513 (11.5) | 0.00 |
| **3** | 939 (8.9) | 359 (8) | -0.04 | 345 (7.8) | 345 (7.8) | 0.00 |
| **Pre-stroke Polypharmacy** | 670 (6.4) | 250 (5.5) | -0.04 | 230 (5.2) | 230 (5.2) | 0.00 |
| **Underlying comorbidity** |  |  |  |  |  |  |
| **Anxiety disorder** | 340 (3.2) | 86 (1.9) | -0.08 | 140 (3.2) | 84 (1.9) | -0.08 |
| **Cancer** | 590 (5.6) | 272 (6) | 0.02 | 212 (4.8) | 269 (6.1) | 0.06 |
| **Chronic back pain** | 4,828 (46) | 854 (18.9) | -0.60 | 1,903 (42.8) | 849 (19.1) | -0.53 |
| **Chronic liver failure** | 333 (3.2) | 116 (2.6) | -0.04 | 135 (3) | 113 (2.5) | -0.03 |
| **COPD** | 252 (2.4) | 145 (3.2) | 0.05 | 114 (2.6) | 144 (3.2) | 0.04 |
| **Cardiovascular disease** | 1,128 (10.7) | 430 (9.5) | -0.04 | 474 (10.7) | 421 (9.5) | -0.04 |
| **Dementia** | 655 (6.2) | 404 (8.9) | 0.10 | 273 (6.1) | 386 (8.7) | 0.10 |
| **Depressive disorder** | 360 (3.4) | 93 (2.1) | -0.08 | 126 (2.8) | 93 (2.1) | -0.05 |
| **Diabetes mellitus** | 1,769 (16.8) | 662 (14.7) | -0.06 | 638 (14.4) | 646 (14.5) | 0.01 |
| **Hyperlipidemia** | 678 (6.5) | 200 (4.4) | -0.09 | 226 (5.1) | 197 (4.4) | -0.03 |
| **Hypertension** | 4,905 (46.7) | 1,953 (43.2) | -0.07 | 1,996 (44.9) | 1,924 (43.3) | -0.03 |
| **Osteoarthritis** | 3,602 (34.3) | 745 (16.5) | -0.42 | 1,367 (30.8) | 731 (16.5) | -0.34 |
| **Osteoporosis** | 839 (8) | 197 (4.4) | -0.15 | 268 (6) | 196 (4.4) | -0.07 |
| **Rheumatic arthritis** | 140 (1.3) | 33 (0.7) | -0.06 | 46 (1) | 33 (0.7) | -0.03 |
| **Renal failure** | 118 (1.1) | 69 (1.5) | 0.04 | 49 (1.1) | 65 (1.5) | 0.03 |
| **Schizophrenia** | 18 (0.2) | 11 (0.2) | 0.02 | 2 (0.1) | 11 (0.3) | 0.05 |

**Supplementary Table 6. Sensitivity analyses on the risk of falls and deaths from all causes in Korean Medicine treatment users compared to non-users in polypharmacy, hyper-polypharmacy, and non-polypharmacy cohorts, further adjusted for prespecified comorbidities.**

|  | **aHR** |
| --- | --- |
| **Polypharmacy after stroke onset**^1^ | |
| **Falls** | |
| KM users (ref. non-users) | 1.45 (1.25 - 1.69) |
| **Death** | |
| KM users (ref. non-users) | 0.72 (0.65 - 0.81) |
| **Subgroup (Hyper-polypharmacy after stroke onset**^2^**)** | |
| **Falls** | |
| KM users (ref. non-users) | 1.11 (0.56 - 2.19) |
| **Death** | |
| KM users (ref. non-users) | 0.73 (0.47 - 1.14) |
| **Sensitivity analysis (Non-polypharmacy after stroke onset**^3^**)** | |
| **Falls** | |
| KM users (ref. non-users) | 1.22 (1.07 - 1.39) |
| **Death** | |
| KM users (ref. non-users) | 0.79 (0.72 - 0.88) |

^1^Polypharmacy after stroke onset: defined as five or more medications prescribed for more than 270 days during the 1-year window after stroke onset

^2^ Hyper-polypharmacy after stroke onset: defined as ten or more medications prescribed for more than 270 days during the 1-year window after stroke onset

^3^ Non-polypharmacy after stroke onset: defined as less than five medications prescribed for more than 270 days during the 1-year window after stroke onset

KM: Korean Medicine treatment; aHR: adjusted hazard ratio

**Supplementary Table 7. Sensitivity analyses on the risk of falls and deaths from all causes in Korean Medicine treatment users compared to non-users in polypharmacy, hyper-polypharmacy, and non-polypharmacy cohorts, further adjusted for medication counts.**

|  | **aHR** |
| --- | --- |
| **Polypharmacy after stroke onset**^1^ | |
| **Falls** | |
| KM users (ref. non-users) | 1.45 (1.25 - 1.69) |
| **Death** | |
| KM users (ref. non-users) | 0.72 (0.67 - 0.77) |
| **Subgroup (Hyper-polypharmacy after stroke onset)** ^2^ | |
| **Falls** | |
| KM users (ref. non-users) | 1.12 (0.56 - 2.24) |
| **Death** | |
| KM users (ref. non-users) | 0.72 (0.46 - 1.14) |
| **Sensitivity analysis (Non-polypharmacy after stroke onset)** ^3^ | |
| **Falls** | |
| KM users (ref. non-users) | 1.23 (1.08 - 1.40) |
| **Death** | |
| KM users (ref. non-users) | 0.77 (0.70 - 0.85) |

^1^Polypharmacy after stroke onset: defined as five or more medications prescribed for more than 270 days during the 1-year window after stroke onset

^2^ Hyper-polypharmacy after stroke onset: defined as ten or more medications prescribed for more than 270 days during the 1-year window after stroke onset

^3^ Non-polypharmacy after stroke onset: defined as less than five medications prescribed for more than 270 days during the 1-year window after stroke onset

KM: Korean Medicine treatment; aHR: adjusted hazard ratio

**Supplementary Table 8. Sensitivity analyses on the interaction between KM use and medication counts in the risk of falls and deaths from all causes in stroke patients.**

|  | **aHR** | ***p*-value** |
| --- | --- | --- |
| **Stroke (I60, 61, 63)** | |  |
| **Falls** | |  |
| KM users (ref. non-users) at 0-4 medication counts | 1.37 (1.13 - 1.66) | 0.844 |
| KM users (ref. non-users) at 5-9 medication counts | 1.40 (1.27 - 1.53) |  |
| KM users (ref. non-users) at 10-14 medication counts | 1.42 (1.12 - 1.81) |  |
| KM users (ref. non-users) at 15-19 medication counts | 1.45 (0.95 - 2.23) |  |
| **Death** | |  |
| KM users (ref. non-users) at 0-4 medication counts | 0.77 (0.66 - 0.90) | 0.526 |
| KM users (ref. non-users) at 5-9 medication counts | 0.73 (0.68 - 0.79) |  |
| KM users (ref. non-users) at 10-14 medication counts | 0.70 (0.57 - 0.85) |  |
| KM users (ref. non-users) at 15-19 medication counts | 0.66 (0.47 - 0.94) |  |

KM: Korean Medicine treatment; aHR: adjusted hazard ratio

**Supplementary Table 9. Incidence rate ratio (IRR) in the cohort before matching (n = 25,034).**

| **Outcome** | **KM use** | **Number of patients** | **Incidence** | **Person-years** | **Incidence rate (IR))** | **IR per 100 person-years** | **IRR** |
| --- | --- | --- | --- | --- | --- | --- | --- |
| **Falls** | No | 7719 | 817 | 19207.29 | 0.04254 | 4.2536 | 1.40 |
|  | Yes | 17315 | 2647 | 44518.33 | 0.05946 | 5.9459 |  |
| **Deaths** | No | 7719 | 1696 | 20370.42 | 0.08326 | 8.3258 | 0.60 |
|  | Yes | 17315 | 2425 | 48407.27 | 0.0501 | 5.0096 |  |
